# Presenteeism and absenteeism: Implications from a study of job insecurity

**DOI:** 10.1002/1348-9585.12158

**Published:** 2020-08-02

**Authors:** Tomohiro Ishimaru

**Affiliations:** ^1^ Department of Environmental Epidemiology Institute of Industrial Ecological Sciences University of Occupational and Environmental Health Japan

This issue of *J Occup Health* published an original article “Job insecurity is associated with presenteeism, but not with absenteeism: A study of 19 720 full‐time waged workers in South Korea” by Kim et al This study has two objectives: to evaluate the relationship between perceived job insecurity and presenteeism or absenteeism and to determine the numbers of days per year of presenteeism and absenteeism that best predict perceived job insecurity.^1^ This study showed that perceived job insecurity was associated with presenteeism of 2 days or more in a year in full‐time waged workers, but no association was observed with absenteeism at any number of days. This means that presenteeism is a marker of workers’ perception of the security of their jobs rather than absenteeism. Additionally, this study provides insights into the cutoff value for days of presenteeism in this field.

The discussion in this article concerns the gap between presenteeism and absenteeism. The authors explained that insecure workers did not take sick leave out of fear of dismissal.^1^ Heponiemi et al reported that subjective job insecurity contributed more to presenteeism than contractual job insecurity, such as fixed‐term employment.^2^ Although these results are carefully assessed in relation to the healthy worker effect because of the cross‐sectional design,^3^ the finding shows an important aspect in understanding the mechanisms underlying presenteeism and absenteeism.

Another discussion point in this article is the cutoff value for days of presenteeism. We previously reported that there is no standard metric for measuring presenteeism, leading to varying definitions in the literature.^4^ The current study used “sickness presenteeism,” which is normally assessed by the number of days per year that employees worked despite feeing unwell.^5^ Because of the lack of a standard value for “sickness presenteeism,” occupational health practitioners cannot define it uniformly in daily practice. Therefore, the findings of the current study, 2 days or more per year of presenteeism, can be used to define “sickness presenteeism” in practice and in future research.

## CONFLICT OF INTEREST

None declared.
